# Dexmedetomidine versus remifentanil for controlled hypotension under general anesthesia: A systematic review and meta-analysis

**DOI:** 10.1371/journal.pone.0278846

**Published:** 2023-01-17

**Authors:** Ning Xu, Linmu Chen, Lulu Liu, Wei Rong

**Affiliations:** 1 Department of Anesthesiology, Weihai Central Hospital affiliated to Qingdao University, Wendeng, Weihai, Shandong, People’s Republic of China; 2 Department of Pain Medicine, Weihai Central Hospital affiliated to Qingdao University, Wendeng, Weihai, Shandong, People’s Republic of China; 3 Department of Respiratory and Critical Care Medicine, Weihai Central Hospital affiliated to Qingdao University, Wendeng District, Weihai, Shandong, People’s Republic of China; Beth Israel Deaconess Medical Center / Harvard Medical School, UNITED STATES

## Abstract

This meta-analysis aimed to analyze and compare the efficacy and safety of remifentanil and dexmedetomidine applied respectively for controlled hypotension under general anesthesia. We searched the Cochrane Library, PubMed, EMBASE, Web of Science, CNKI, SinoMed, Wanfang, and VIP databases, as well as dissertations and conference papers, to obtain randomized controlled trials comparing remifentanil and dexmedetomidine applied respectively for controlled hypotension before August 23, 2021. The primary outcomes included hemodynamic profiles, surgical field score, and blood loss. Extubation time, sedation and pain score at the PACU, and perioperative adverse events were the secondary outcomes. Nine randomized controlled trials with 543 patients (272 in the dexmedetomidine group and 271 in the remifentanil group) were eventually included. This meta-analysis indicated no significant difference between dexmedetomidine and remifentanil in terms of surgical field score, blood loss, minimum values of mean arterial pressure (MD 0.24 with 95% CI [-1.65, 2.13], P = 0.80, I^2^ = 66%) and heart rate (MD 0.42 [-1.33, 2.17], P = 0.64, I^2^ = 40%), sedation scores at the PACU (MD -0.09 [-0.69, 0.50], P = 0.76, I^2^ = 92%), and incidence of bradycardia (OR 2.24 [0.70, 7.15], P = 0.17, I^2^ = 0%). Compared with remifentanil, dexmedetomidine as the controlled hypotensive agent showed a lower visual analogue score at the PACU (MD -1.01 [-1.25, -0.77], P<0.00001, I^2^ = 0%) and incidence of shivering (OR 0.22 [0.08, 0.60], P = 0.003, I^2^ = 0%), nausea, and vomiting (OR 0.34 [0.13, 0.89], P = 0.03, I^2^ = 0%). However, extubation time was shorter in the remifentanil group (MD 3.34 [0.75, 5.93], P = 0.01, I^2^ = 90%). In conclusion, dexmedetomidine and remifentanil are both effective in providing satisfactory controlled hypotension and surgical conditions. Dexmedetomidine is better in easing postoperative pain at the PACU and reducing the occurrence of shivering, nausea, and vomiting. Meanwhile, remifentanil is a fast-track anesthesia with a shorter extubation time. Given the limitations of this meta-analysis, further studies are needed for a more definitive comparison of the efficacy and safety of dexmedetomidine and remifentanil.

## Introduction

Cushing first proposed controlled hypotension (CH) in 1917, the magnitude of which could be defined as a reduction of the systolic blood pressure to 80–90 mmHg, a reduction of mean arterial pressure (MAP) to 50–65 mmHg or a 30% reduction of baseline MAP [1, 2]. This anesthetic technique has been practiced for decades to facilitate surgery and to reduce bleeding and transfusion requirement [1, 3]. CH is frequently performed with pharmacological agents. Some of these agents can be used successfully not only alone, but also in combination treatment to mitigate the side effects of each other [2]. Remifentanil is an ultra-short-acting opioid with a distinct pharmacokinetic profile, clinical versatility, and improved control of its action, making it one of the primary agents that has been successfully used alone for CH [4, 5]. Remifentanil has demonstrated benefits in terms of decreasing MAP and bleeding, and it is widely used for CH in various surgical procedures such as open rhinoplasty, lumbar laminectomy, and other pentacameral operations [3, 6, 7]. Dexmedetomidine, an α_2_-adrenoceptor agonist, is another commonly used agent in CH with sedative, anxiolytic, sympatholytic, and analgesics paring effects, and minimal depression of respiratory function [8]. Previous research indicates that its role as a controlled hypotensive agent in general anesthesia is comparable to other commonly used CH techniques and capable of providing a favorable surgical field condition [1, 9, 10]. Moreover, Zhang et al. [11] found that combination treatment of dexmedetomidine and remifentanil has a better controlled hypotensive effect, higher quality of recovery from anesthesia, and lower occurrence of adverse effects than either drug alone.

One of the current topical issues concerning CH during surgery is to find the surgical patients’ characteristics and advantages/disadvantages of controlled hypotensive agents for selecting the appropriate agents in clinical practice [12]. Remifentanil and dexmedetomidine are commonly used to compare with other drugs to better guide controlled hypotensive agent selection [13–15]. A previous meta-analysis compared dexmedetomidine with magnesium and showed that the advantages of selecting dexmedetomidine for CH included promising surgical field condition, favorable CH, and minimized use of opioid or analgesia administration [16]. Alkan et al. [12] found that the advantages of remifentanil for CH included less volatile agent, shorter time to achieve CH, stable blood pressure, lower surgical field bleeding scores, and longer duration with the targeted MAP when compared to nitroglycerin. Recently, there have been some studies of CH comparing remifentanil with dexmedetomidine. Some debate subsequently occurred concerning remifentanil and dexmedetomidine in the selection of controlled hypotensive agents. Menshawi et al. [17] reported that patients treated with dexmedetomidine for CH had better quality and more extended postoperative analgesia than did those with remifentanil. Additionally, Karabayirli et al. [18] and Zamani et al. [19] demonstrated that dexmedetomidine for CH is of limited value as it is associated with less pronounced effect of reducing intraoperative bleeding, longer postoperative anesthesia recovery, and higher postoperative sedation scores when compared with remifentanil. However, currently, no relevant literature provides systematic evidence in terms of comparing the two agents in CH. Therefore, this study aimed to investigate the efficacy and safety of remifentanil and dexmedetomidine used for CH under general anesthesia, as well as to discover the advantages of the two agents for better guidance of controlled hypotensive agent selection in clinical practice.

## Methods

Our meta-analysis complied with the recommendations in the Preferred Reporting Items for Systematic Reviews and Meta Analyses (PRISMA) statement [20] and the guidelines described in the Cochrane Handbook.

### Search strategy

We searched the Cochrane Library, PubMed, EMBASE, Web of Science, SinoMed, CKNI, WanFang, and VIP databases, as well as dissertations and conference papers, from inception to August 23, 2021. Published randomized controlled clinical trials (RCTs) of dexmedetomidine versus remifentanil for CH under general anesthesia were collected. The keywords used were “remifentanil,” “dexmedetomidine,” “controlled hypotension,” and “general anesthesia”. The flowchart of the PubMed search strategy as an example is depicted in S1 Fig.

### Selection criteria

Inclusion criteria of this study were listed below:

1. Study design: Only RCTs.
2. Participants: Surgical patients who received dexmedetomidine or remifentanil for CH under general anesthesia.
3. Intervention: Dexmedetomidine as the controlled hypotensive agent for the intervention group (D group).
4. Comparison: Remifentanil as the controlled hypotensive agent for the control group (R group).
5. Outcomes: Clinical evaluation of controlled hypertensive agents mainly focused on facilitating hemodynamic profiles and surgical conditions; thus, we perceived hemodynamic indicators (MAP; heart rate, HR) and surgical condition indicators (surgical field score and blood loss) as primary outcomes. Surgical field score was tested according to the Fromme scale (S1 Table) [21]. Blood loss was measured uniformly in mL. Secondary outcomes included extubation time, sedation and pain scores (visual analogue scale, VAS) in the PACU, and safety indicators (incidence of perioperative adverse events). Extubation time referred to the time interval between the end of surgery and tracheal extubation.

The exclusion criteria were as follows: (1) non-randomized controlled trials, (2) literature reports of poor quality and ineligibility, (3) failure to provide original data, (4) duplicate literature or crossover of study subjects, and (5) similar reports or incomplete information.

### Study selection and data extraction

Two authors conducted a computer search independently, and the included literature was then checked for duplicates. The titles and abstracts of the remaining literature were read in accordance with the inclusion and exclusion criteria. Following the exclusion of trials that clearly did not meet the inclusion criteria, full texts of trials that might meet the inclusion criteria were read to see if those were eventually included. For the studies that met the inclusion criteria, the data were extracted independently by two reviewers according to a data extraction form developed in advance, which included the following: (i) basic information about the included literature, including title, author, year, and contact information; (ii) methodological characteristics, such as the method of generating random sequences, blinding, etc.; and (iii) general characteristics of the study population, including age, sex, sample size, and specific details of outcome for both groups. Incomplete data were supplemented by contacting the authors by email or other means. Two authors cross-checked the results of the included trials, and differing reviews were addressed by discussion with a third reviewer.

### Study quality assessment and statistical analysis

The quality of evidence of the included studies was evaluated in strict accordance with the Cochrane Risk of Bias Assessment Tool [22] in terms of the method of generating randomized group sequences, allocation concealment, blinding of the participants and outcome assessment, incomplete data, selective reporting, and other bias. The overall risk of bias was divided into three categories: ‘Low’ for RCTs with a low risk of bias in all domains, ‘High’ for RCTs with a high risk of bias in at least one domain, and "Unclear" for RCTs with neither ‘Low’ nor ‘High’ risk of bias.

Review Manager 5.4 software was used for statistical analysis. For continuous variables, the data were counted, and the mean difference (MD) was applied to the 95% confidence interval (CI). For dichotomous variables, odds ratios (OR) with 95% CI were calculated. The I^2^ and Chi-square tests were used to assess heterogeneity among included studies. The degree of heterogeneity was calculated in conjunction with the I^2^ value. When there is no statistical heterogeneity among the studies (I^2^<50%), a fixed-effects model was then used; when there was significant heterogeneity among the studies (I^2^>50%), a random-effects model was used. Sensitivity analysis was performed to explore the sources of heterogeneity when necessary. A subgroup analysis based on different time points was performed. In terms of publication bias, we used Stata15.1 software to perform Egger’s and Begg’s tests, then presented the Egger’s funnel plot and related details. A P<0.05 for each test was considered to indicate obvious publication bias.

## Results

### Search results

**Fig 1:**
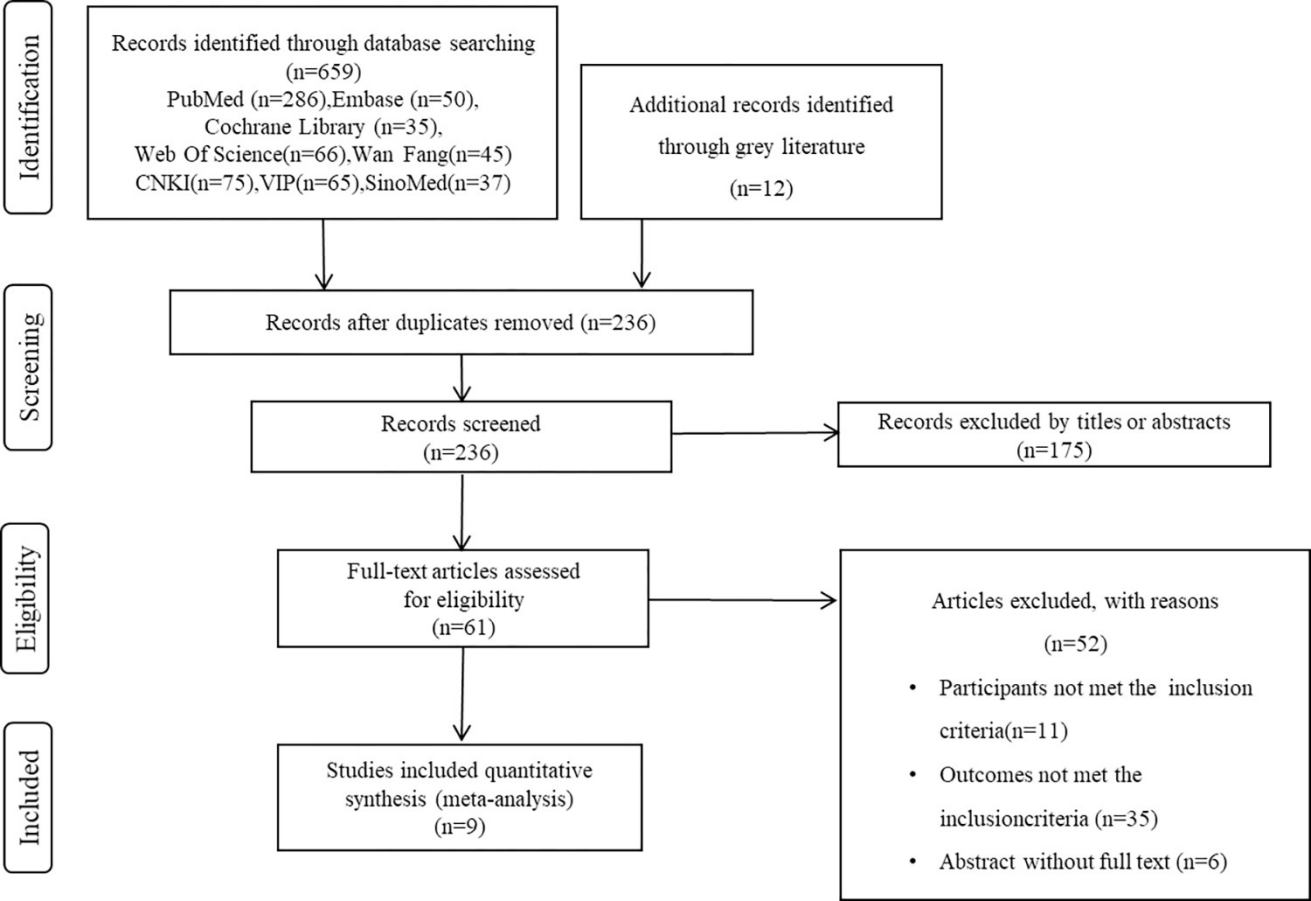
Flowchart of the literature search and screening process.

Fig 1 summarizes the literature search and screening process. A total of 671 relevant articles were identified in accordance with the literature search strategy and screening methods; 236 studies were deleted after screening out duplicates with Endnote X9 software, and 175 studies were excluded after reading the titles and abstracts. Finally, nine articles were chosen for this meta-analysis after excluding those that did not meet the inclusion criteria and provided insufficient text.

### Risk of bias assessment

**Fig 2:**
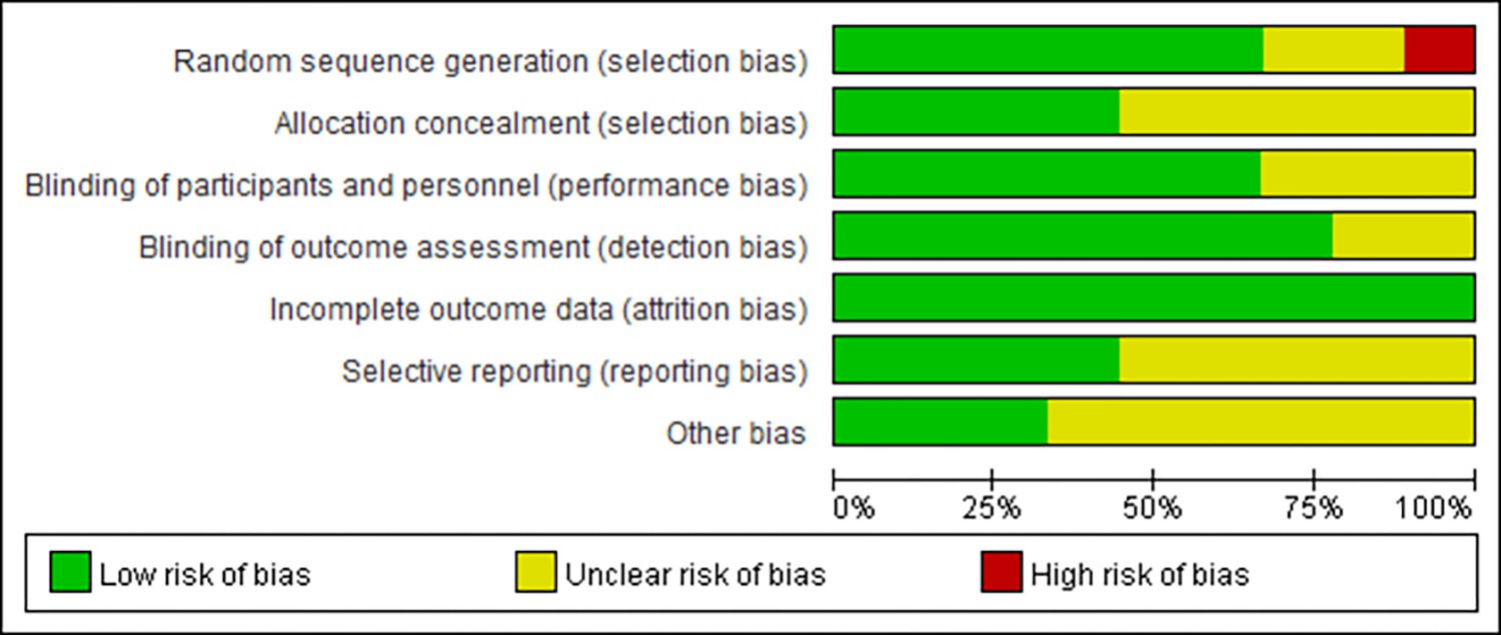
Risk of bias summary in RCTs.

**Fig 3:**
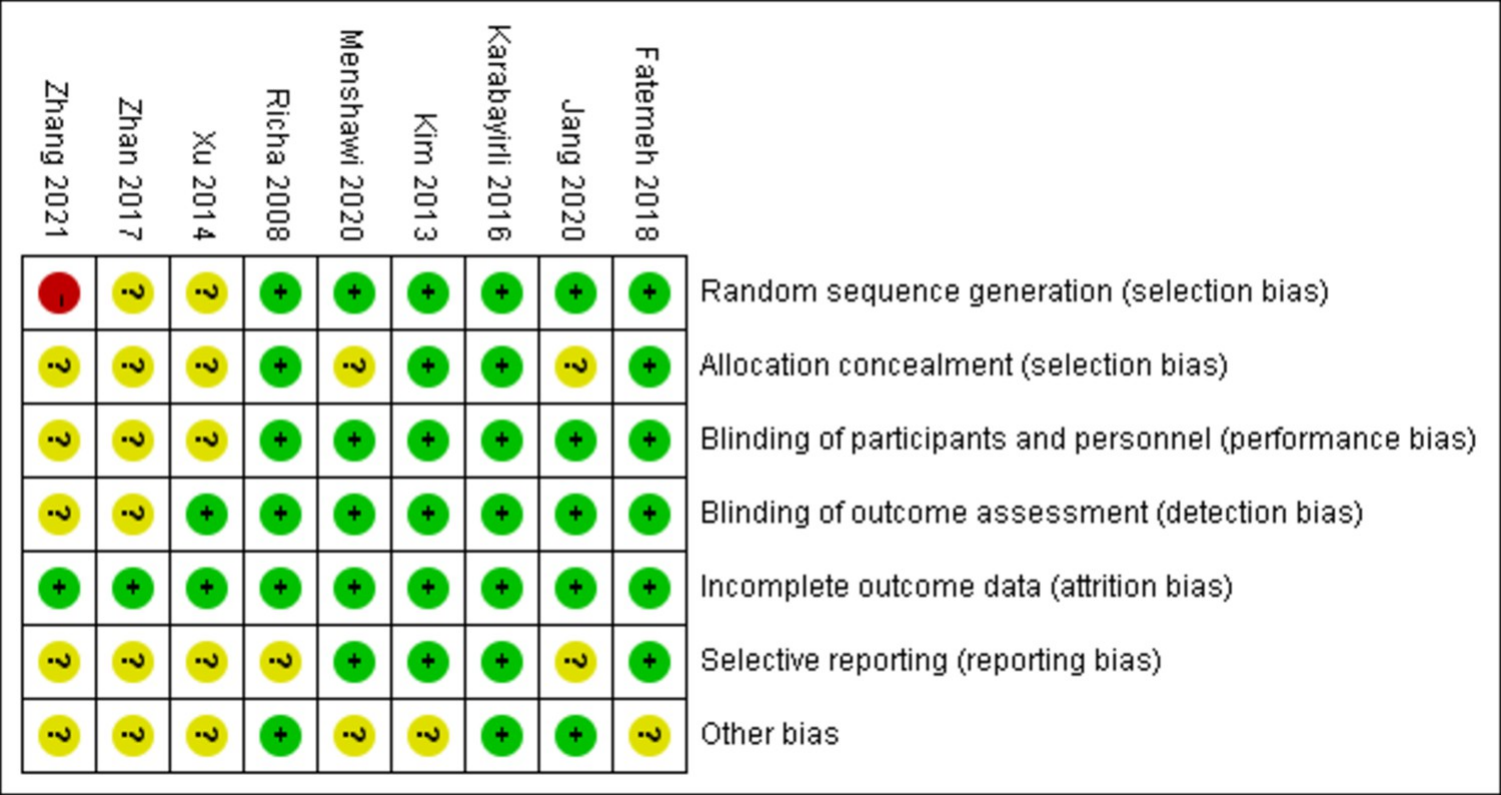
Risk of bias graph in RCTs.

We generated figures (Figs 2 and 3) demonstrating the risk of bias for the included studies. Six studies [5, 17, 18, 23, 24, 27] described random generation methods in detail, two studies [25, 26] only mentioned random, and one study’s [11] random method had a high risk. Four of the nine studies [5, 18, 23, 24] had a low risk of bias for allocation concealment, while the others had an unclear risk of bias. Six studies [5, 17, 18, 23, 24, 27] meticulously documented blinding of participant, personnel, and outcome assessment. All of the included studies provided complete outcome data with low attrition bias. Most of the included studies were categorized as ‘Low’ or ‘Unclear’ in terms of selecting reporting and other bias.

### Study characteristics

Basic information concerning the included studies are listed in Table 1. Nine RCTs that met the criteria were included. Six of the nine included studies were in English from five countries and three were in Chinese, with a total of 543 patients (272 in the dexmedetomidine group and 271 in the remifentanil group). Five types of procedures requiring CH were identified, including endoscopic sinus surgery, lumbar discopathy surgery, tympanoplasty, shoulder arthroscopy, and total knee arthroscopy. The follow-up period was not mentioned in six studies.

**Table 1:** Basic information concerning the included studies.

Author, year	Country	Sample(D/R)	Age		Surgery time		Surgery type	Intervention		Ref.	Outcome
			D group	R group	D group	R group		D group	R group		
**Kim, 2013**	Korea	32/34	45.5±12.1	39.8±17.4	61.19±26.6	65.12±23.05	Endoscopic sinus surgery	Dexmedetomidine 1 μg/kg over 10 min followed by a continuous infusion of 0.4–0.8 μg/kg/h.	Remifentanil hydrochloride 1 μg/kg over 1 min followed by a continuous infusion of 0.2–0.4 μg/kg/min	[5]	**Ⅰ, Ⅱ, Ⅲ, Ⅳ, Ⅵ, Ⅶ**
**Zhang, 2021**	China	35/31	71.89±2.74	72.33±2.54	__	_	Total knee arthroscopy	Dexmedetomidine 0.8 μg/kg over 10 min, followed by a continuous infusion of 0.5 μg/kg/hr	Remifentanil initial dose of 0.1 μg/kg/min, followed by an increase of 0.1 μg/kg/min every 2 min.	[11]	**I, IX**
**Menshawi, 2020**	Egypt	20/20	40.66±8.14	42.73±9.62	147.84±21.57	139.77±18.93	Shoulder arthroscopy	Dexmedetomidine 1 μg/kg over 10 min followed by a continuous infusion of 0.3–0.6 μg/kg/h	Remifentanil 1 μg/kg over 1 min followed by a continuous infusion of 0.25–0.5 μg/kg/min	[17]	**Ⅰ, Ⅱ, Ⅴ, Ⅵ, Ⅶ, Ⅸ**
**Karabayirli, 2016**	Turkey	23/24	37±14.1	36±13.3	98±80.7	105±48.2	Endoscopic sinus surgery	Dexmedetomidine 1 lg/kg over 10 min, followed by a continuous infusion of 0.7 lg/kg/h	Remifentanil 1 μg/kg over 1 min followed by a continuous infusion of 0.25–0.5 μg/kg/min	[18]	**IV**
**Fatemeh, 2018**	Iran	30/30	36.57±8.04	37.97±9.58	46.3±4.72	50.19±4.91	Lumbar discopathy surgery	Dexmedetomidine, 0.3–0.7 μg/kg/h, plus a continuous infusion of propofol, 50–100 μg /kg/min	Remifentanil, 0.1–1 μg/kg/h, plus a continuous infusion of propofol, 50–100 μg /kg/min	[23]	**Ⅳ, Ⅸ**
**Richa, 2008**	Lebanon	12/12	34.2±9.6	36.6±9.9	123.33±35.25	107.75±32.97	Tympanoplasty	Dexmedetomidine 1 μg/kg/h over 10 min followed by a continuous infusion of 0.4–0.8 μg/kg/h	Remifentanil 1 μg /kg/h over 1 min followed by a continuous infusion of 0.2–0.4 μg /kg/min	[24]	**Ⅰ, Ⅱ, Ⅲ, Ⅴ**
**Xu, 2014**	China	20/20	38±11	40±13	100.3±11.7	97.4±7.6	Endoscopic sinus surgery	Dexmedetomidine 1 μg/kg over 10 min followed by a continuous infusion of 0.2–0.7 μg/kg/h	Remifentanil 1 μg/kg over 1 min followed by a continuous infusion of 0.2–0.4 μg/kg/min	[25]	**Ⅰ, Ⅱ, Ⅲ, Ⅴ, Ⅵ, Ⅶ, Ⅳ**
**Zhan, 2017**	China	30/30	37.4±13.1	38.4±12.3	90±23	88±25	Endoscopic sinus surgery	Dexmedetomidine 1 μg/kg over 10 min followed by a continuous infusion of 0.2–0.7 μg/kg/h	Remifentanil 1 μg/kg over 1 min followed by a continuous infusion of 0.2–0.4 μg/kg/min	[26]	**I, II, III, IV, V**
**Jang, 2020**	Korea	70/70	46±17	42±17	35±25	31±17	Endoscopic sinus surgery	Dexmedetomidine 1 μg/kg over 10 min, followed by a continuous infusion of 0.4–0.8 μg/kg/h	Remifentanil 1 μg/kg over 1 min, followed by a continuous infusion of 0.2–0.4 μg/kg/min.	[27]	**Ⅳ, Ⅴ, Ⅵ, Ⅶ**

Ⅰ, mean arterial pressure; Ⅱ, Heart rate; Ⅲ, surgical field score; Ⅳ, blood loss; Ⅴ, extubation time; Ⅵ, sedation score at the PACU; Ⅶ, VAS at the PACU; Ⅸ, perioperative adverse events; “-”, without detailed information; D or D group, dexmedetomidine group; R or R group, remifentanil group

### Results of this meta-analysis

All the results of this meta-analysis are summarized in Table 2.

**Table 2:** Result summaries of this meta-analysis.

Outcomes		OR/MD (95% CI)	Heterogeneity test			Overall effect test		Egger’s test
			Q-value	P-value	I^2^	Z-value	P-value	P-value
**I**		0.24 [-1.65, 2.13]	14.78	0.01	0.66	0.25	0.80	0.358
**II**		0.42 [-1.33, 2.17]	10.06	0.12	0.40	0.47	0.64	0.422
**III**		-0.03 [-0.39, 0.33]	11.70	0.008	0.74	0.17	0.86	0.486
**IV**		4.07 [-7.95, 16.09]	1.28	0.94	0	0.66	0.51	0.877
**V**		3.34 [0.75, 5.93]	48.91	<0.00001	0.90	2.52	0.01	-
**VI**	**Upon arrival at the PACU**	-0.09 [-1.21, 1.03]	72.27	<0.00001	0.96	0.16	0.87	-
	**30 min after arrival at the PACU**	-0.12 [-1.05, 0.81]	15.63	0.0004	0.87	0.25	0.80	-
	**60 min after arrival at the PACU**	-0.03 [-1.99, 1.93]	12.47	0.0004	0.92	0.03	0.98	-
	**Total (95% CI)**	-0.09 [-0.69, 0.50]	100.81	<0.00001	0.92	0.31	0.76	-
**VII**	**Upon arrival at the PACU**	-0.99 [-1.35, -0.64]	0.00	0.98	0	5.45	<0.00001	-
	**30 min after arrival at the PACU**	-1.13 [-1.58, -0.69]	0.20	0.66	0	4.98	<0.00001	-
	**60 min after arrival at the PACU**	-0.89 [-1.36, -0.42]	0.27	0.60	0	3.73	0.0002	-
	**Total (95% CI)**	-1.01 [-1.25, -0.77]	1.02	0.96	0	8.24	<0.00001	-
**IX**	**shivering**	0.22 [0.08, 0.60]	0.08	0.77	0	2.96	0.003	-
	**Nausea and vomiting**	0.34 [0.13, 0.89]	0.31	0.85	0	2.20	0.03	-
	**Bradycardia**	2.24 [0.70, 7.15]	0.20	0.66	0	1.36	0.17	-
	**Total (95% CI)**	0.51 [0.23, 1.14]	10.20	0.12	0.41	1.64	0.10	-

Ⅰ, mean arterial pressure; Ⅱ, Heart Rate; Ⅲ, surgical field score; Ⅳ, blood loss; Ⅴ, extubation time; Ⅵ, sedation scores at the PACU; Ⅶ, VAS at the PACU; Ⅸ, perioperative adverse events; OR, odds ratio; MD, mean difference; CI, confidence interval.

### Comparison of efficacy on hemodynamic indicators

**Fig 4:**
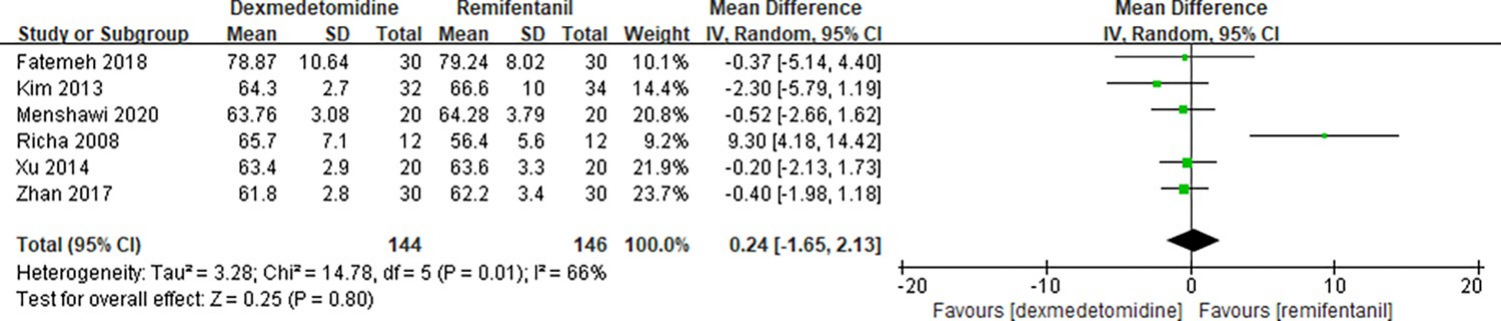
Forest plot of effects on MAP. Dexmedetomidine group vs. remifentanil group.

**Fig 5:**
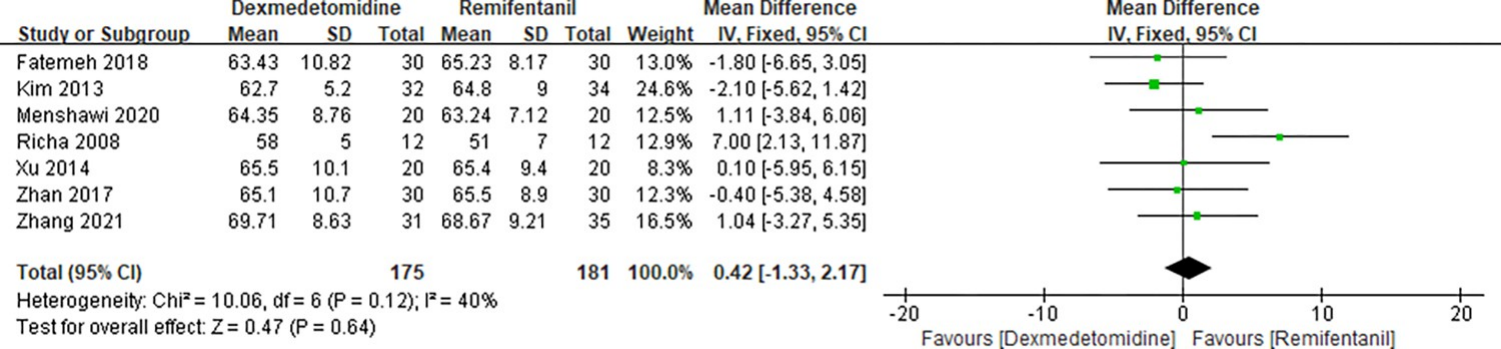
Forest plot of effects on HR. Dexmedetomidine group vs. remifentanil group.

Since the relevant hemodynamic parameters in our incorporated RCTs were measured repeatedly during controlled hypotensive administration or depicted intricately in a diagram, we identified and analyzed the minimum values of the hemodynamic responses (MAP, HR) in the meta-analysis and the related data were extracted if accessible in the original article. Six studies [5, 17, 23–26] evaluated the MAP minimum values during CH period and the results of this meta-analysis showed that there was no significant difference between dexmedetomidine and remifentanil (MD 0.24 with 95% CI [-1.65, 2.13], P = 0.80, I^2^ = 66%) (Fig 4). Meanwhile, seven studies [5, 11, 17, 23–26] evaluated the HR minimum values during CH period. The forest plot of the results are presented in Fig 5. There was no significant difference between dexmedetomidine and remifentanil (MD 0.42 with 95% CI [-1.33, 2.17], P = 0.64, I^2^ = 40%).

### Comparison of efficacy on surgical condition indicators

**Fig 6:**
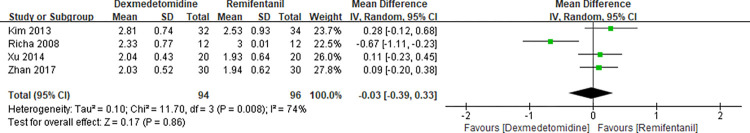
Forest plot of surgical field score. Dexmedetomidine group vs. remifentanil group.

**Fig 7:**
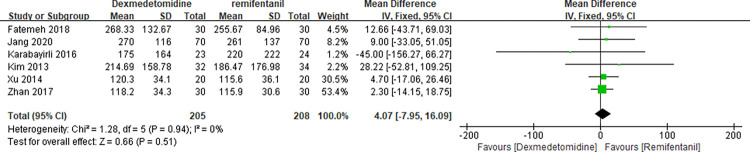
Forest plot of blood loss. Dexmedetomidine group vs. remifentanil group.

Four RCTs [5, 24–26] reported surgical field scores between the two groups. This meta-analysis (Fig 6) presented no significant differences between dexmedetomidine and remifentanil (MD -0.03 with 95% CI [-0.39, 0.33], P = 0.86, I^2^ = 74%). Six studies tested blood loss between the two groups.[5, 18, 23, 25–27] There was no significant difference between dexmedetomidine and remifentanil (MD 4.07 with 95% CI [-7.95, 16.09], P = 0.51, I^2^ = 0%) (Fig 7).

### Extubation time

**Fig 8:**
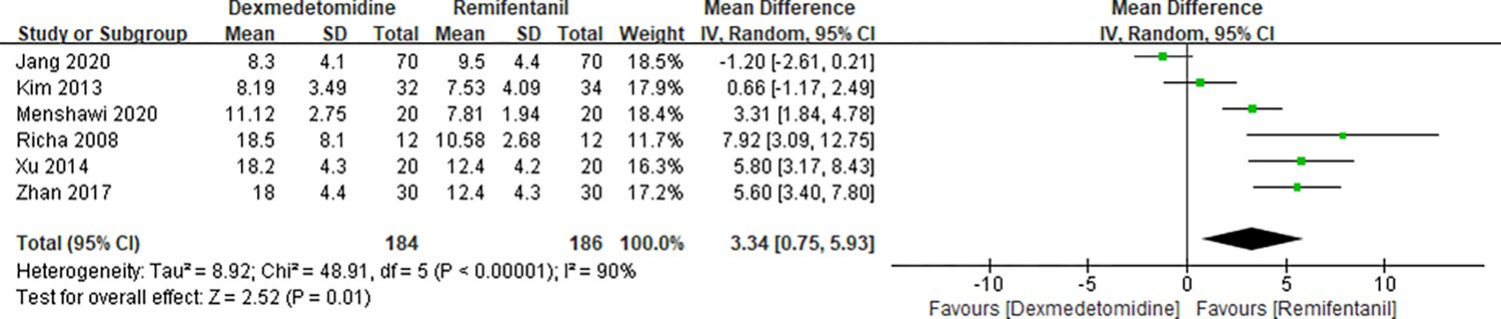
Forest plot of extubation time. Dexmedetomidine group vs. remifentanil group.

Six RCTs [5, 17, 24–27] tested extubation time of the two groups. This meta-analysis showed a significant difference between the two groups (MD 3.34 with 95% CI [0.75, 5.93], P = 0.01, I^2^ = 90%) (Fig 8).

### Sedation scores at the PACU

**Fig 9:**
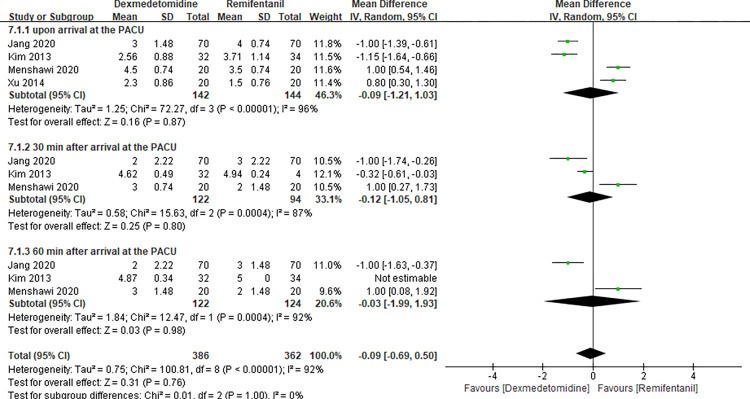
Forest plot of sedation scores at the PACU. Dexmedetomidine group vs. remifentanil group.

Four studies [5, 17, 25, 27] evaluated the sedation score at the PACU and the results of this meta-analysis are presented in Fig 9. There was no significant difference between dexmedetomidine and remifentanil (MD -0.09 with 95% CI [-0.69, 0.50], P = 0.76, I^2^ = 92%). We conducted a subgroup analysis according to three different time points (upon arrival at the PACU, and 30 min and 60 min after arrival at the PACU). No significant differences were found at the three different time points.

### VAS at the PACU

**Fig 10:**
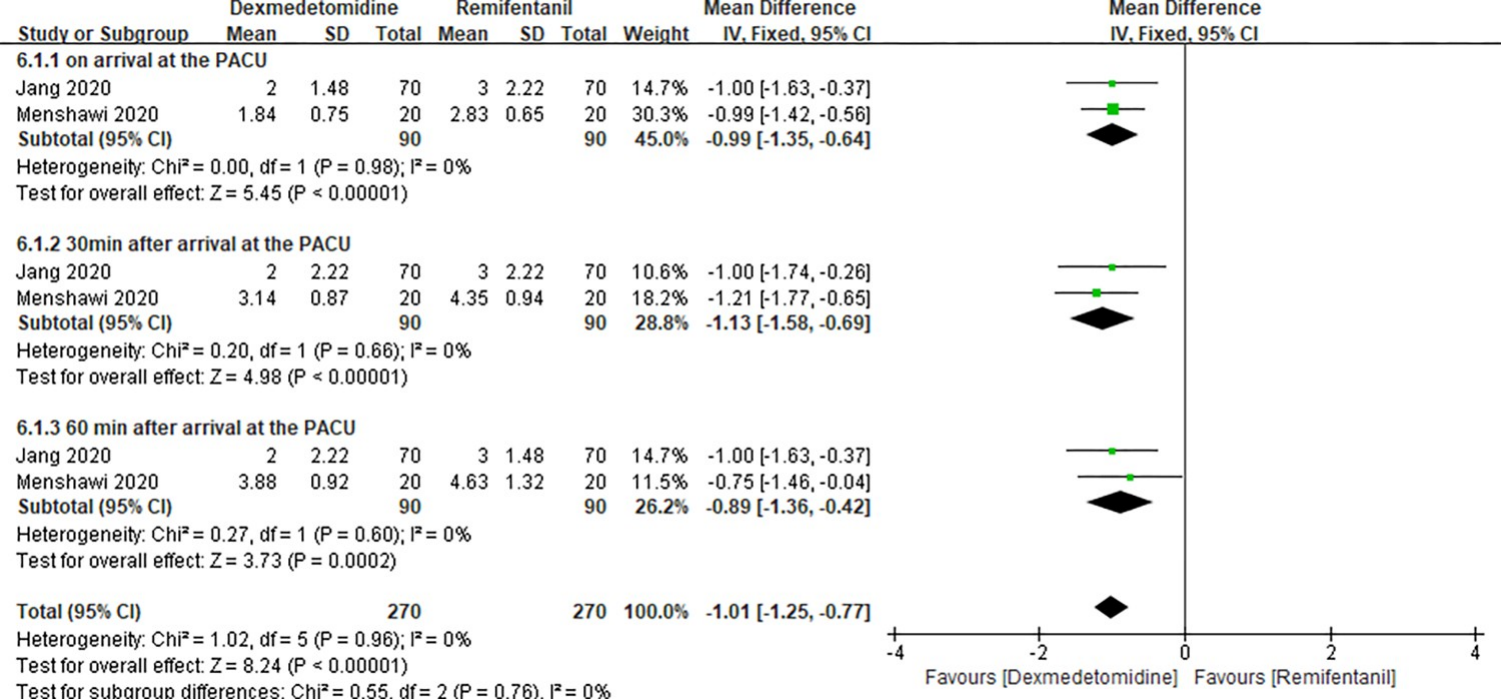
Forest plot of VAS at the PACU. Dexmedetomidine group vs. remifentanil group.

Two studies [17, 27] evaluated the VAS at the PACU, and this meta-analysis showed a significant difference between dexmedetomidine and remifentanil (MD -1.01 with 95% CI [-1.25, -0.77], P<0.00001, I^2^ = 0%) (Fig 10). Further subgroup analysis was performed, the results of which showed a significant difference at the three different time points.

### Comparison on safety indicators

**Fig 11:**
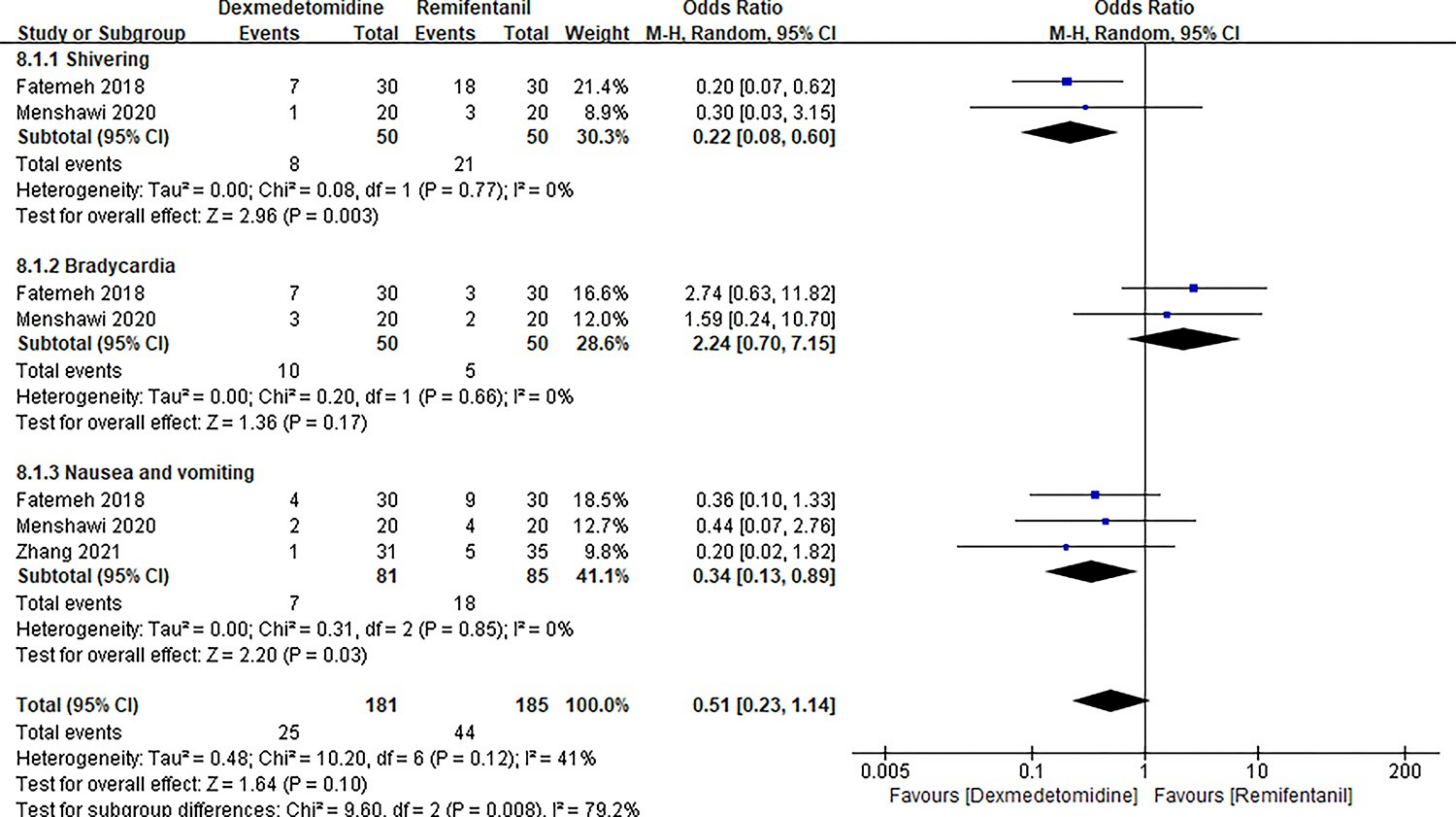
Forest plot of perioperative adverse events. Dexmedetomidine group vs. remifentanil group.

Three studies [11, 17, 23] mentioned perioperative adverse events of nausea and vomiting (Fig 11). Two studies [17, 23] documented perioperative adverse events of shivering and bradycardia. We conducted a subgroup analysis according to three adverse events (shivering, nausea and vomiting, bradycardia). As a result, there was no significant difference on the occurrence of bradycardia (OR 2.24 with 95% CI [0.70, 7.15], P = 0.17, I^2^ = 0%). However, compared with the remifentanil group, there were lower occurrence of shivering (OR 0.22 with 95% CI [0.08, 0.60], P = 0.003, I^2^ = 0%), nausea, and vomiting (OR 0.34 with 95% CI [0.13, 0.89], P = 0.03, I^2^ = 0%) in the dexmedetomidine group. The results of this meta-analysis indicated that there was no significant difference between dexmedetomidine and remifentanil in overall adverse events (OR 0.51 with 95% CI [0.23, 1.14], P = 0.10, I^2^ = 41%) (Fig 11).

### Sensitivity analysis

After conducting the meta-analysis, we found significant heterogeneity (I^2^>50%) across the four domains: MAP, surgical field score, extubation time, and sedation scores at the PACU. To explore the sources of heterogeneity, a sensitivity analysis was conducted. Sensitivity analysis revealed that heterogeneity of MAP (MD -0.52 with 95% CI [-1.52, 0.4], P = 0.30, I^2^ = 0%) and surgical field score (MD 0.14 with 95% CI [-0.05, 0.33], P = 0.16, I^2^ = 0%) were significantly reduced after removing Richa’s study [24]. Additionally, three studies [5, 17, 27] may be the source of heterogeneity in the extubation time domain. After deleting these three papers, I^2^ was zeroed (MD 5.93 with 95% CI [4.33, 7.52], P<0.00001, I^2^ = 0%). Concerning sedation scores at the PACU, sensitivity analysis cannot reduce the significant heterogeneity in this domain.

### Publication bias

We run the Egger’s and Begg’s test for primary outcomes to investigate publication bias. The P-value for the Egger’s test is shown in Table 2, and the S1 File displays the Egger’s publication bias plot as well as the details of the two tests. Based on the results of the Egger’s and Begg’s tests, the P-value of the Egger’s and Begg’s tests was higher than 0.05. This indicates no evidence of significant publication bias among primary outcomes. However, due to the small number of trials included in each domain, Egger’s and Begg’s test for primary outcomes may be influenced by a type II random error.

## Discussion

We conducted the first systematic review and meta-analysis that analyzed dexmedetomidine versus remifentanil for CH under general anesthesia. This current meta-analysis depicted that there was no statistically significant difference in terms of hemodynamic indicators (MAP and HR), surgical condition indicators (surgical field score and blood loss), sedation score at the PACU, and the incidence of bradycardia between dexmedetomidine and remifentanil. However, the advantage of the dexmedetomidine group was statistically significant in terms of VAS at the PACU and the occurrence of shivering, nausea, and vomiting. Compared with the dexmedetomidine group, extubation time of the remifentanil group was significantly shorter.

Six studies assessed the minimum intraoperative MAP value, and the results of this meta-analysis revealed no significant difference between dexmedetomidine and remifentanil with high heterogeneity [5, 17, 23–26]. It is concerning that the studies conducted by Richa et al. [24] only included 24 participants, while the number of participants included in other studies was also insufficient. Thus, there may be potential bias associated with the RCT design, which could have a significant impact on the current meta-analysis’s results. A sensitivity analysis confirmed that the heterogeneity in the field was caused by this study and that removing the study significantly reduced the heterogeneity.

Seven studies including 356 participants evaluated the intraoperative minimum values of HR [5, 11, 17, 23–26]. A previous study showed that remifentanil was associated with better intraoperative hemodynamic stability, which produced minimal alterations of arterial blood pressure and HR in doses up to 2 μg/kg [28, 29]. Dexmedetomidine is an α2-adrenoceptor agonist, its infusion of which results in an increase in blood pressure combined with a marked decrease in HR due to α2 -receptor activation in vascular smooth muscles. Moreover, α2-receptor activation leads to peripheral vasoconstriction and thereby hypertension accompanied by a quick reduction in HR, probably caused by the baroceptor reflex [30, 31]. Nevertheless, our meta-analysis indicated that there was no significant difference between dexmedetomidine and remifentanil.

Meanwhile, four RCTs evaluated surgical field score [5, 24, 25], and six mentioned blood loss [5, 18, 23, 25–27]. These two outcomes are frequently used to assess surgical conditions. This meta-analysis found no significant difference between dexmedetomidine and remifentanil in surgical conditions, which was consistent with Hwang’s study [9]. Furthermore, a previous meta-analysis found that dexmedetomidine is an effective method for producing CH in a surgical field with favorable conditions [16]. Alkan et al. discovered that remifentanil provides a lower surgical field bleeding score when compared to other agents such as esmolol and nitroglycerin [12]. The current meta-analysis cannot prove which of dexmedetomidine and remifentanil is superior, but previous studies have shown that both dexmedetomidine and remifentanil can provide satisfactory surgical conditions [12, 16].

We tested the extubation time of the two groups [5, 17, 24–27] and concluded that remifentanil had a shorter extubation time after CH. The conclusion of the present meta-analysis was consistent with the results of the enrolled RCTs [8, 9]. Fast-track anesthesia is an anesthetic technique that facilitates rapid postoperative recovery for patients and has been applied in adult congenital heart disease surgery in many countries [32]. The advantage of fast-track anesthesia is the early return of spontaneous ventilation and extubation. Kim et al. discovered that patients treated with remifentanil for CH recovered faster than those treated with dexmedetomidine in the immediate postoperative period [9]. Remifentanil undergoes widespread extravascular metabolism and is rapidly metabolized via extrahepatic, nonspecific blood and tissue esterases, which contributes to its rapid clearance [4]. Remifentanil’s pharmacokinetics allow for an early return of spontaneous ventilation and extubation [33]. Furthermore, Menshawi et al. [17] reported that patients given dexmedetomidine had a longer postoperative anesthesia recovery than those given remifentanil [34]. We identified a significant heterogeneity in the results of this meta-analysis, which can also be resolved by sensitivity analysis after deleting three papers [5, 17, 27].

We analyzed the sedation score at the PACU, and the results of this meta-analysis indicated that there was no significant difference between dexmedetomidine and remifentanil. The following subgroup analysis came to the same conclusion. Although Menshawi et al. [17] and Jang et al. [27] revealed that dexmedetomidine for CH had better postoperative sedative and analgesic effects than remifentanil, the calculated data from the current meta-analysis is not powered enough to support the advantage of dexmedetomidine in sedation. Notably, obvious heterogeneity, which may result from different types of surgery, methods of anesthesia induction, or a lack of powered RCTs, cannot be addressed by conducting the sensitivity analysis.

Another outcome we used to conduct this meta-analysis and subgroup analysis is VAS at the PACU. We found that dexmedetomidine showed a better analgesic effect than remifentanil at the PACU. Similar results were found in the subgroup analysis based on three different timepoints. The two included studies demonstrated that the dose of dexmedetomidine infusion fluctuated from 0.3 to 0.8 μg/kg/h [9, 16]. A previous study has shown that a 0.4 μg/kg dose of dexmedetomidine, as a sole analgesic, can be effectively used for pain relief after laparoscopic tubal ligation, although possibly accompanying undesirable side effects during the recovery period [35]. The analgesic effectiveness of dexmedetomidine is controversial. In Tugba’s research, analgesic effect of dexmedetomidine was recognized as it was associated with better analgesia and lower postoperative pain score than intravenous anesthetic agent (propofol) in patients undergoing hysteroscopic surgery [36]. At the same, Menshawi et al. [17] and Jang et al. [27] showed that patients in the dexmedetomidine group had better extended postoperative analgesia compared with opioid analgesic (remifentanil). However, Kim et al. [5] found that the present study demonstrated that there were no significant differences in postoperative pain between the two groups. From the meta-analysis results, dexmedetomidine was better in easing postoperative pain without obvious heterogeneity. However, the results of this meta-analysis are likely to be influenced by future studies due to the small sample size of the enrolled studies.

Perioperative shivering, nausea, and vomiting, as well as bradycardia, are common complications after general anesthesia [37–39]. Currently, these adverse events have been reported in all kinds of studies to test the safety of different agents for CH. A previous meta-analysis reported that the use of dexmedetomidine is associated with increased risk of bradycardia, while dexmedetomidine and remifentanil shared the similar risk of bradycardia from the current [16]. Our meta-analysis suggested less occurrence of shivering, nausea, and vomiting in the dexmedetomidine group compared with the remifentanil group. Despite the results of the meta-analysis, the current data cannot draw authoritative conclusion due to the shortage of sufficiently powered RCT.

The current meta-analysis has some strengths. First, this is the first systematic review and meta-analysis to compare dexmedetomidine to remifentanil for CH during general anesthesia. Second, we used sensitivity analysis to reduce the high heterogeneity of most domains. Finally, to investigate publication bias, we used the Egger’s and Begg’s test for primary outcomes, and the results revealed no obvious bias. However, there are a few limitations to this meta-analysis that should be addressed. Our meta-analysis included a small number of high-quality RCTs and participants, which may not result in authoritative conclusions. In addition, there was significant clinical heterogeneity in four domains due to different dexmedetomidine and remifentanil dosages, different types of surgery, and inconsistent outcome measurement methods. Study designs and outcome measurements should be agreed upon to improve the quality of future comparisons between dexmedetomidine and remifentanil under general anesthesia.

## Conclusion

Dexmedetomidine and remifentanil are both effective in providing satisfactory CH and surgical conditions. However, dexmedetomidine is better in easing postoperative pain at the PACU and reducing the occurrence of shivering, nausea, and vomiting. Meanwhile, remifentanil is a fast-track anesthesia with a shorter extubation time. Given the limitations of our meta-analysis, further studies are still needed for a more definitive conclusion on the comparison of the efficacy and safety of dexmedetomidine and remifentanil.

## Supporting information

S1 Checklist PRISMA checklist 2020. (DOCX)

S1 Fig Flowchart of the PubMed search strategy. (TIF)

S1 Table Fromme scale of surgical field score. (DOCX)

S1 File Publication bias. (A) Results of the Begg’s test and Egger’s test. (B) Egger’s publication bias plot.
(DOCX)
