# Supplementary figures and images for: Dexmedetomidine versus remifentanil for controlled hypotension under general anesthesia: A systematic review and meta-analysis
Source: PLoS One. 2023 Jan 17;18(1):e0278846. doi: 10.1371/journal.pone.0278846 (PMC9844847; doi:10.1371/journal.pone.0278846)

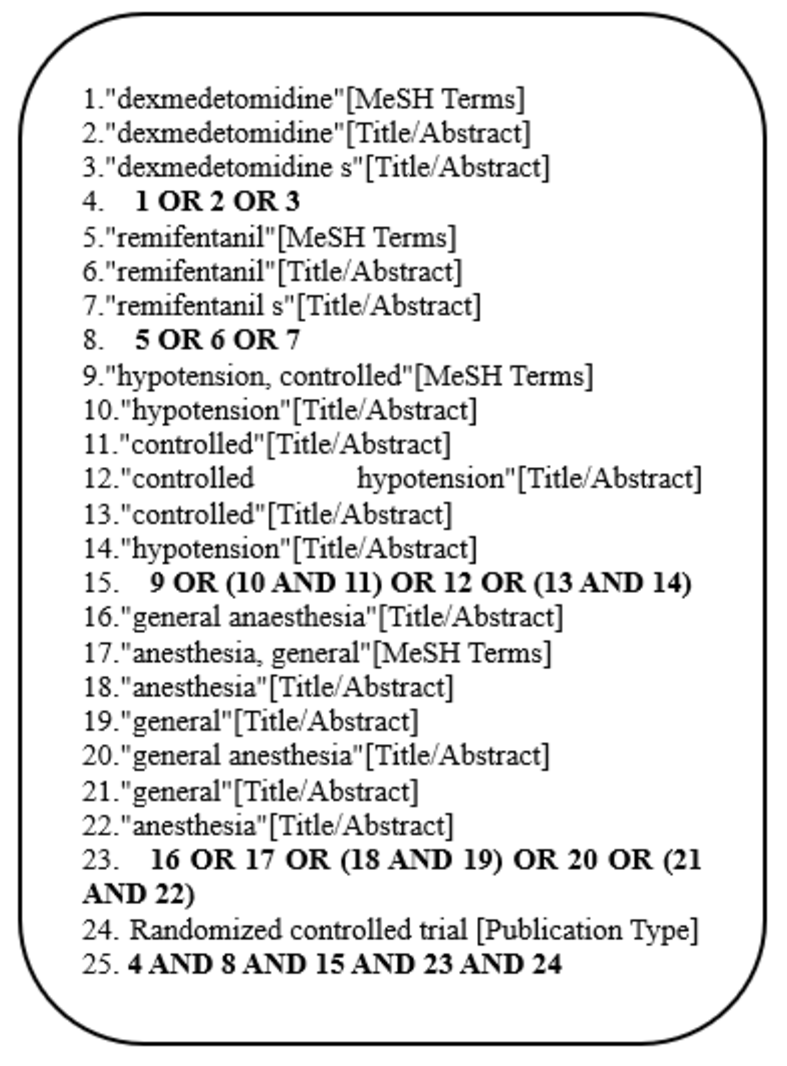

Supplement: S1 Fig — (TIF) [file pone.0278846.s002.tif]
