# Supplementary material for: Dexmedetomidine versus remifentanil for controlled hypotension under general anesthesia: A systematic review and meta-analysis
Source: PLoS One. 2023 Jan 17;18(1):e0278846. doi: 10.1371/journal.pone.0278846 (PMC9844847; doi:10.1371/journal.pone.0278846)
Supplement: S1 Table — (DOCX) [file pone.0278846.s003.docx]

S1 Table 1. **The Fromme scale of surgical field score**

| **The Fromme scale** |  | **Specific items** |
| --- | --- | --- |
| **0** |  | No bleeding |
| **1** |  | Minor bleeding in the surgical field, no aspiration required; |
| **2** |  | Minor bleeding in the surgical field, need for aspiration on occasion, but may obstruct the surgical field |
| **3** |  | Slight bleeding in the surgical field, requiring frequent suctioning and bleeding again a few seconds after stopping suctioning, obstructing the surgical field |
| **4** |  | Moderate bleeding in the surgical field, requiring frequent suctioning and obstructing the surgical field if suctioning is stopped |
| **5** |  | Severe bleeding in the surgical field necessitating continuous suctioning, as well as bleeding obstructing the surgical field |
