# Supplementary material for: Dexmedetomidine versus remifentanil for controlled hypotension under general anesthesia: A systematic review and meta-analysis
Source: PLoS One. 2023 Jan 17;18(1):e0278846. doi: 10.1371/journal.pone.0278846 (PMC9844847; doi:10.1371/journal.pone.0278846)
Supplement: S1 File — (A) Results of the Begg’s test and Egger’s test. (B) Egger’s publication bias plot. (DOCX) [file pone.0278846.s004.docx]

a.

b.

**S1 File 1: publication bias for MAP** a:results of Begg’s test and Egger’s test;b: Egger’s publication bias plot

a.

b.

**S1 File 2: publication bias for HR** a:results of Begg’s test and Egger’s test;b: Egger’s publication bias plot

a.

b.

**S1 File 3: publication bias for surgical field score.** a:results of Begg’s test and Egger’s test;b: Egger’s publication bias plot

a.

b.

**S1 File 4: publication bias for blood loss** a:results of Begg’s test and Egger’s test;b: Egger’s publication bias plot
